# Indicators of loss of consciousness in slaughter without stunning: a neuroanatomical reappraisal

**DOI:** 10.3389/fvets.2026.1838183

**Published:** 2026-05-07

**Authors:** Jacob R. Hascalovici, Ruth Woiwode, Hyman M. Schipper, Joane Parent

**Affiliations:** 1 Department of Neurology, Hackensack Meridian Neuroscience Institute, Hackensack Meridian School of Medicine, Nutley, NJ, United States; 2 Saul R. Korey Department of Neurology, Albert Einstein College of Medicine, Bronx, NY, United States; 3 The Arthur S. Abramson Department of Physical Medicine and Rehabilitation, Bronx, NY, United States; 4 Department of Anesthesiology, Bronx, NY, United States; 5 Department of Animal Science, University of Nebraska-Lincoln, Lincoln, NE, United States; 6 Department of Neurology and Neurosurgery, Lady Davis Institute, Jewish General Hospital, McGill University, Montreal, QC, Canada; 7 Faculté de Médecine Vétérinaire, Université de Montréal, Saint-Hyacinthe, QC, Canada

**Keywords:** cattle, cerebral cortex, halal, loss of consciousness (LOC), shechita, slaughter without stunning

## Abstract

Minimizing animal suffering during slaughter depends on appropriate pre-slaughter handling, technique, and post-slaughter assessment. The post-slaughter phase must include rapid determination of loss of consciousness (LOC) using scientifically based indicators. In religious slaughter, pre-slaughter stunning is prohibited by Jewish law and generally opposed under Islamic law. Consciousness and pain perception arise from complex interactions within the cerebral cortex. International standards governing assessment of LOC commonly rely on indicators of brainstem failure. This approach is appropriate in conventional slaughter with stunning, a technique designed to cause traumatic injury to the cerebral cortex and brainstem. In contrast, religious slaughter is intended to induce LOC through slaughter by exsanguination via ventral neck (SEVNI) where rapid cerebral hypoperfusion results in loss of cortical function. Accordingly, reliance on cortical rather than brainstem indicators is more consistent with the underlying neurobiology of LOC in religious slaughter. In this paper, we examine the neuroanatomical basis of commonly used indicators of (un)consciousness in cattle and assess their scientific and practical applicability to religious slaughter. Brainstem-based indicators have limited applicability in the context of SEVNI, as they do not account for the specific neurophysiological mechanisms underlying LOC in this setting. The current approach is also subject to substantial inter-observer variability and introduces avoidable occupational safety hazards. While not a behavioral indicator, visual confirmation of continuous, high-volume blood flow from the transected carotid arteries reflects rapid cerebral hypoperfusion and is physiologically consistent with irreversible, near-instantaneous LOC. This assessment can be performed reliably from a safe distance.

## Introduction

**Figure 1:**
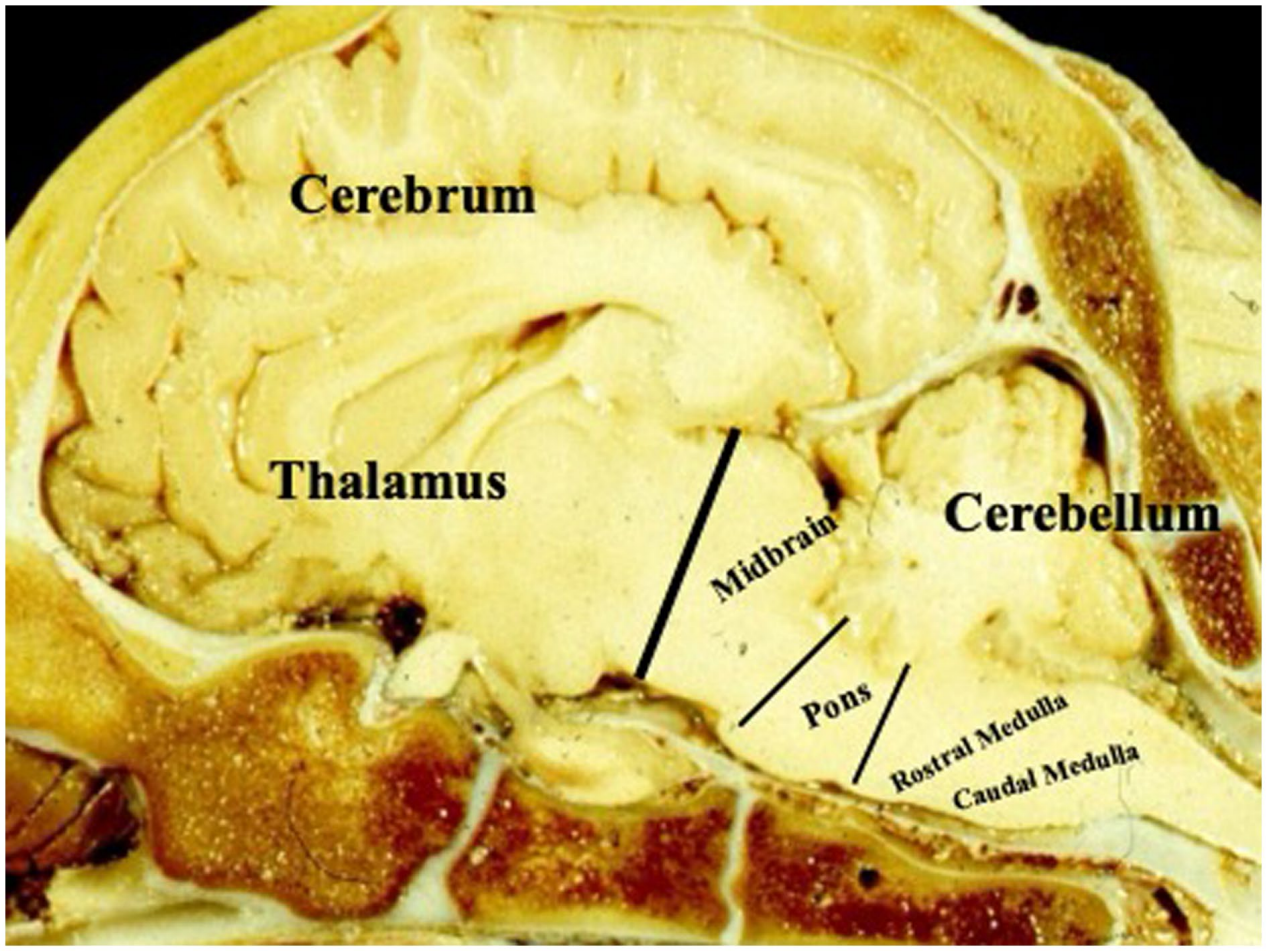
Paramedian view of a ruminant (ovine) brain displaying the different parts of the brain.

**Figure 2:**
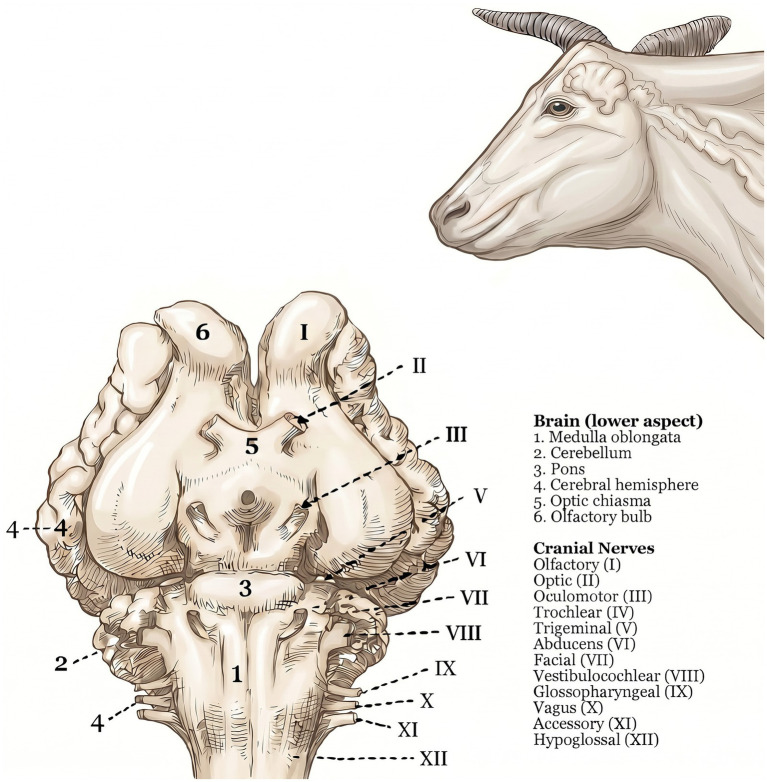
Illustration of the ventral aspect of the bovine brainstem depicting the cranial nerves (https://bodyofelements.com/products/cow-nervous-system-anatomy-poster-18-x-24).

The bovine brain is structurally similar to the human brain (1). Both have a brainstem, cerebellum, and cerebral cortex (2–4) (Figure 1). In cattle, as in humans, the brainstem contains the cranial nerve nuclei III–XII, with the corresponding cranial nerves exiting at various levels to perform their respective functions (2, 4) (Figure 2). In humans, the cerebral cortex performs a vast number of interconnected functions, including but not limited to behavioral and emotional control, decision-making, pain perception and consciousness (5). Although our knowledge of the functional correlations between the cattle and human brains is limited, it is well recognized that the cerebral cortex of domestic species is responsible for consciousness and goal-directed behaviors. Importantly, motor control in domestic species relies more heavily on extrapyramidal, primarily brainstem-mediated pathways than in primates, such that preservation of certain reflexive motor outputs does not necessarily imply preserved cortical function (4, 6). Consistent with this, in practice, inspection personnel and plant operators are trained to disregard reflexive motor activity, such as kicking or paddling, when assessing sensibility (7). Cortical function depends critically on uninterrupted cerebral blood flow and perfusion pressure to sustain tissue oxygenation and is therefore especially vulnerable to anoxia (8, 9). By contrast, although the brainstem is also dependent on adequate tissue oxygenation, it exhibits substantially greater resilience to anoxic conditions (10, 11). Cortical *function*, which reflects integrated communication among multiple brain regions to generate higher-order cognitive processes, is distinct from cortical *activity*, which refers to electrical signaling in individual neurons or local circuits (12–14) that may persist even without consciousness and can be observed under anesthesia when consciousness is absent (15).

Animals can experience positive and negative affective states such as pain, pleasure and stress, all of which are processed in the cerebral cortex (16). Animal pain is defined as “an aversive feeling or sensation associated with actual or potential tissue damage and resulting in physiologic, neurophysiologic, neuroendocrine, and behavioral changes that indicate a stress response” (17, 18). The perception of pain is cognitively complex and entirely dependent on consciousness, which in turn requires a functional cerebral cortex (19, 20). In cattle, the transmission of pain signals (nociception) functions much as it does in humans. Peripheral nerve fibers convert pain-related messages (noxious stimuli) into electric impulses that travel to the spinal cord where signals are directed to the thalamus and onward to other relevant brain areas within the cerebral cortex for further processing (17). It is important to note that in the central nervous system, nociception is the cortical activity associated with noxious stimuli and can therefore proceed in the absence of cortical function such as during unconsciousness. This is because the signals sent as a part of the pain sensing pathway are distinct from the pathways and functions of pain processing (21). Stated succinctly, nociception involves neural signal transmission, while pain perception involves the interpretation and conscious awareness of these signals (22).

Conventional cattle slaughter includes pre-slaughter stunning to render the animal unconscious prior to exsanguination, most commonly using a captive bolt. Loss of consciousness (LOC) following captive bolt stunning is understood to result from a traumatic injury to both the cortical and brainstem structures. Because the extent of neural tissue injury cannot be directly observed, operators must rely on either behavioral indicators to assess stun effectiveness and confirm that LOC has occurred. In Islamic practice, Halal slaughter (Dhabīḥah) is the prescribed method for killing animals for dietary consumption and must adhere to specific guidelines. The animal must be in good health, and a sharp knife must be used to swiftly cut the windpipe, esophagus, and blood vessels in the throat (23). In the Jewish religion, Shechita is the traditional method of slaughter of designated (‘kosher’) mammals and birds for dietary consumption. During Shechita of cattle, a certified worker (*shochet*) uses a surgically sharp, nick-free blade (*chalaf*) to cut the trachea, esophagus, carotid arteries, and jugular veins rapidly and uninterruptedly (24–26). Pre-slaughter stunning is prohibited by Jewish law and is generally opposed under Islamic law (27). Instead, in both Shechita and Dhabīḥah, LOC and death are induced through a single act of slaughter by exsanguination via ventral neck incision (SEVNI). Loss of consciousness following SEVNI is understood to result from rapid cortical failure due to acute cerebral hypoperfusion, secondary to substantial blood loss and the consequent marked reduction in cerebral blood flow and perfusion pressure (28–30). Unlike stunning, which relies on behavior-based indicators of LOC, the effectiveness of Shechita and Halal slaughter can be directly and objectively verified by visual confirmation of continuous, high-volume blood flow from the transected carotid arteries. This finding reflects rapid cerebral hypoperfusion and loss of cerebral perfusion pressure required to sustain cortical function (28, 29) and is therefore physiologically indicative of irreversible, near-instantaneous LOC.

This review presents a critical evaluation of the indicators for LOC. It is guided by the central thesis that LOC in cattle slaughter without stunning is a cortical phenomenon driven by rapid and irreversible cerebral hypoperfusion, and that indicators mediated primarily by brainstem function reflect later stages of neural failure rather than the onset of LOC. On this basis, the review evaluates commonly used indicators according to their underlying neuroanatomical substrates and assesses their scientific validity, practical applicability, and occupational safety implications in the context of religious slaughter.

## Methods

This review is based on a targeted literature search of PubMed, Medline, the Cochrane Library, Google Scholar, and Web of Science focusing on studies relevant to loss of consciousness in cattle during slaughter. Search terms included combinations of “cattle,” “slaughter,” “loss of consciousness,” “electrophysiology,” “exsanguination,” “behavioral indicators,” and “brainstem reflexes.”

### Current global guidelines and the neuroanatomical localization of indicators of (un)consciousness

Table 1 summarizes current recommendations from the leading global institutions on the choice of indicators of (un)consciousness following slaughter without stunning from leading global institutions (31–35).

**Table 1:** Comparison of indicators of consciousness, loss of consciousness (LOC), and brain death across major international regulatory and professional organizations.

Organization	Indicators		
	Consciousness	LOC	Brain death
World Organization for Animal Health (35)	N/A	Absent muscle tone	Cessation of blood flow
		Absent corneal reflex	
		Absent palpebral reflex	
		Loss of rhythmic breathing	
		Tonic–clonic seizure^1^	
		Apnea^1^	
North American Meat Institute (33)	Standing	Absent menace response	Absent corneal reflex
	No loss of posture	Absent palpebral reflex	Absent palpebral reflex
	Righting reflex	Absent corneal reflex	Loss of rhythmic breathing
	Vocalizations	Loss of rhythmic breathing	
	Unprovoked blinking	Loss of posture	
	Menace response	Eye roll	
	Eye tracking		
European Food Safety Authority (34)	No collapse	Cessation of breathing	Cessation of bleeding
	Attempt to regain posture	Absent muscle tone	Relaxed body
	Rhythmic breathing	Loss of posture	Dilated pupils
	Corneal reflex	Absent corneal reflex	
	Palpebral reflex	Absent palpebral reflex	
	Righting reflex	Permanent collapse^1^	
	Eye rotation	Apnea^1^	
	Vocalizations	Seizure^1^	
		Fixed eyes^1^	
		Absent vocalization^1^	
Canadian Food Inspection Agency (32)	Standing posture	Absent rhythmic breathing	Blank stare
	Stiff or curled tongue	Loss of corneal reflex^2^	Dilated pupils
	Vocalizations	Eye roll	Absent corneal reflex
	Spontaneous blinking with eye tracking	Loss of posture	Apnea
	Menace response	Lack of jaw and body tone	Terminal gasps
		Blank stare	Cessation of blood flow
		Absent pupillary response	
		Absent palpebral reflex^**2**^	
		Absent reaction to nose prick	
American Veterinary Medical Association (31)	Standing posture	Absent corneal reflex	Absent corneal reflex
	Head or body righting reflex	Absent rhythmic breathing	Absent rhythmic breathing
	Voluntary vocalizations	Loss of palpebral reflex	Loss of palpebral reflex
	Spontaneous blinking without stimulation	Loss of posture	
	Eye pursuit of moving objects	Loss of righting reflex^**1**^	
	Menace response	Loss of ability to stand and walk	

1 Specific to stunning.

2 Confirmed by three consecutive negative tests 20 s apart.

The table highlights substantial variability in indicator classification and illustrates that the most frequently recommended indicators are brainstem mediated.

#### Absence of rhythmic breathing

The primary rhythm generator for breathing resides in the caudal portion of the brainstem, particularly within the pre-Bötzinger complex and adjacent respiratory centers of the medulla (36). The respiratory control nuclei of the brainstem project to motor neurons in the phrenic nerve (C3–C7 depending on species) that drive the diaphragm and to spinal motor neurons controlling intercostal and accessory respiratory muscles enabling coordinated breathing movements (36). Cortical input can voluntarily modulate breathing, for example during swallowing or breath-holding, but such control is not necessary for rhythmic respiration to occur. In the context of SEVNI, respiratory centers in the brainstem may continue to control breathing, until brainstem ischemia ensues, producing residual gasping or rhythmic movements of the chest or jaw. Consequently, the persistence of breathing does not indicate consciousness, while its absence only reflects failure of brainstem centers which occurs after consciousness has already been abolished. Moreover, accurate assessment of rhythmic or other abnormal breathing patterns is technically challenging, requires knowledge and specific training, and is time-consuming, rendering it impractical in slaughterhouse settings.

#### Loss of posture (LOP)

The bovine stay apparatus is a largely passive system of ligaments and tendons that allows cattle to stand with minimal muscular effort once the limbs are positioned in a mechanically “locked” posture. Although neural control is required to initiate this posture, maintaining it requires little ongoing cortical or voluntary motor activity. Posture is maintained primarily by brainstem structures, including the reticular formation and vestibular nuclei (via reticulospinal, rubrospinal, and vestibulospinal pathways), with modulatory input from the cerebellum and limited influence from the cerebral cortex (4, 6). The brainstem, being more resilient to hypoxia and ischemia, can sustain postural after bleeding due to loss of inhibitory effects on the spinal lower motor neurons (LMN) by the upper motor neuron (UMN) nuclei, primarily localized in the brainstem (6, 37). Consequently, cattle may remain standing after LOC because the passive stay apparatus can continue to support body weight until progressive loss of muscle tone or circulatory collapse leads to physical collapse. Moreover, animal studies have shown that locomotion can return in chronically decerebrated cats emphasizing that the neural circuitry responsible for locomotion in domestic species is located in the brainstem and spinal cord (37). While LOP is a reliable indicator that LOC has already occurred, it is a conservative and delayed marker of LOC. However, it has the practical advantage of being easily recognizable, even from a distance.

#### Righting reflex

The righting reflex, which enables an animal to orient itself back to an upright posture, is an interplay between sensory input, central processing and motor output. Although primarily mediated by the brainstem with key contributions from the vestibular nuclei, reticular formation, superior colliculi, and their descending pathways to the spinal cord (4), it necessitates cortical processing of visual and somatosensory information. In the context of slaughter, residual subcortical and brainstem activity may still generate stereotyped motor outputs such as attempts to right the body (mainly the head and neck), eye movements, or limb jerks. Human studies of rapid-onset syncope have shown that unconscious subjects can still exhibit righting attempts, repetitive oral movements, and ocular reflexes despite LOC, underscoring that such behaviors do not indicate consciousness (38). Indeed, certain reflexive movements, myoclonic jerks or flailing are normally suppressed by an intact functioning cortex and only emerge when cortical control is removed causing brainstem or spinal circuits to become disinhibited (39, 40). Therefore, these movements indicate that cortical function and consciousness have already been lost. Distinguishing righting attempts from reflex motor activity is challenging and often requires close examination by an operator, necessitating proximity to the animal and introducing a non-trivial risk of injury (41).

#### Vocalizing, jaw movements, and tongue stiffness

Jaw, facial, and tongue movements are mediated by brainstem motor nuclei, including the trigeminal motor nucleus (cranial nerve V) in the pons which controls mastication, the facial motor nucleus (cranial nerve VII) in the rostral medulla which controls facial expression, and the hypoglossal nucleus (cranial nerve XII) in the caudal medulla which innervates the intrinsic and extrinsic tongue muscles responsible for tongue movement and tone (2, 3). Although these nuclei receive descending input from the motor cortex via corticobulbar tracts, they are also strongly influenced by the reticular formation and other brainstem circuits capable of generating reflexive, rhythmic, or tonic motor output independent of cortical function (42). As a result, post-slaughter behaviors such as chewing-like jaw movements, apparent vocalizing attempts, tongue stiffness, or sporadic tongue movements can arise from brainstem-mediated reflexes rather than conscious motor control.

#### Corneal/Palpebral/Blink reflex

The corneal reflex is a blink elicited by stimulation of the cornea, the palpebral reflex is a blink triggered by stimulation of the eyelids or periocular region, and the blink reflex refers more broadly to a blink evoked by sensory stimulation. In all cases, afferent input is carried by the trigeminal nerve (cranial nerve V): the ophthalmic branch for the corneal reflex, and the ophthalmic and maxillary branches for the palpebral reflex (2, 3). This sensory information is relayed to the trigeminal sensory nucleus in the medulla. From there, interneurons within the reticular formation connect to the facial motor nucleus (cranial nerve VII) in the rostral medulla, which sends efferent fibers to the orbicularis oculi muscle, producing eyelid closure (2, 4). Because these reflex arcs are mediated entirely within the brainstem, they can persist irrespective of cortical function and are only abolished with the onset of brainstem ischemia and hypoxia. Thus, in slaughter settings, absence of the corneal and palpebral reflexes signifies loss of brainstem function and therefore progression to death, making them uninformative for determining the onset of LOC.

#### Eye tracking

Eye tracking (the ability to follow a moving object) in domestic animals involves a complex, multi-level system that depends on widespread cortical, subcortical and brainstem integration to process visual, vestibular, and proprioceptive inputs. The system is characterized by laterally positioned eyeballs, a high percentage of decussation of the optic nerve (> 80%), and reliance on brainstem reflexes for gaze stabilization. The key structures are the optic pathways by way of the geniculate nucleus and optic tectum for visual processing and orientation, and projections to the brainstem which send impulses through the medial longitudinal fasciculus to oculomotor (III), trochlear (IV) and abducens (VI) nerves for tracking when the head is stationary. This test is impractical because an anxious, highly stressed but neurologically normal animal may exhibit a fixed gaze. In addition, distinguishing purposeful from non-purposeful eye movements is difficult even under controlled conditions, making the test unreliable and potentially unsafe in the slaughterhouse setting (43).

#### Loss of menace response

**Figure 3:**
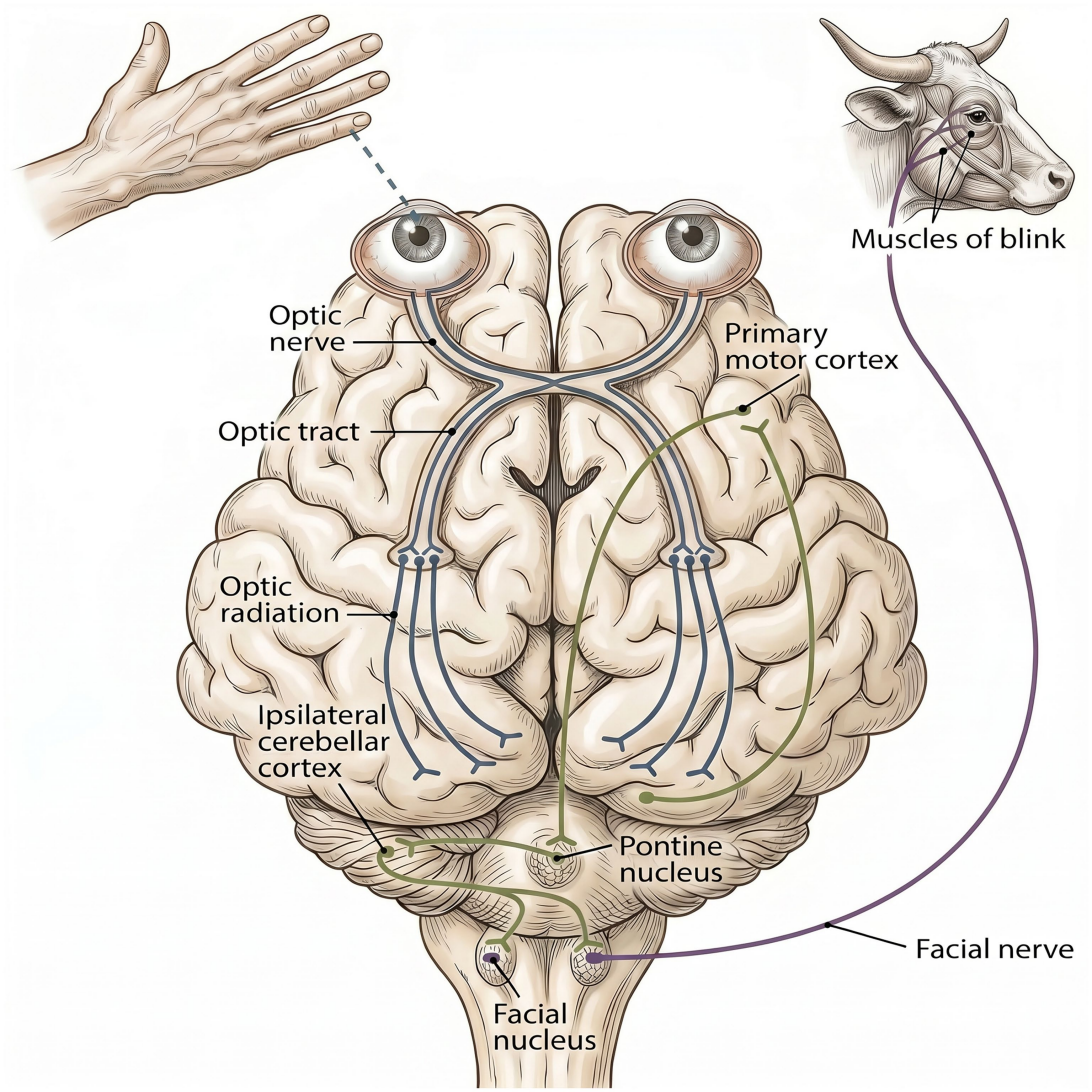
Diagram illustrating the mammalian (canine) afferent portion of the menace response [visual pathway (blue and grey)] and the efferent neural pathways making up the menace response (green and purple) (71).

The menace response is a blink in response to a threatening hand movement directed at one eye while observing for closure of the eyelids. It evaluates the visual pathways, the facial nerve and the cerebellum. Since it requires intact cortical processing, it is a response and not a reflex. Visual input is detected by the retina and transmitted through the optic nerve (cranial nerve II) to the lateral geniculate nucleus of the thalamus, to the occipital lobe. From there, signals are relayed through the visual cortex and motor cortex which integrate the stimulus and generate a descending motor command. This cortical output travels to interneurons in the brainstem reticular formation. Clinical experience indicates cerebellar involvement, although a definite pathway has not been identified. It is postulated that cerebellar coordination facilitates the activation of the facial motor nucleus (cranial nerve VII) located in the medulla which subsequently leads to contraction of the orbicularis oculi muscle and closure of the eyelid (6) (Figure 3). Since this response (or indicator) depends critically on functional cortical and subcortical circuits, not just brainstem integrity, the presence of a menace response indicates preserved cortical function and therefore conscious awareness. This test is greatly influenced by the animal state of mind. Anxiety, fear, or stress often leads to absent menace responses limiting its practical utility in the slaughterhouse setting. Moreover, differentiating a true menace response from reflexive eye blinking is challenging even under controlled conditions and requires close operator proximity to the animal, raising occupational safety concerns (41, 43).

## Discussion

This review evaluates the neuroanatomical basis of commonly used behavioral indicators of LOC in cattle slaughter. In doing so, it identifies a potential misalignment between several currently recommended indicators and the neurobiological processes underlying LOC following SEVNI. Specifically, prevailing guidelines for cattle slaughter without stunning rely predominantly on indicators reflecting brainstem (dys)function which correspond more closely to later stages of neural failure and progression to brain death rather than cortical failure. This approach is scientifically coherent in conventional slaughter where stunning causes a traumatic injury to cortical and brainstem structures. In contrast, this approach does not adequately account for the fundamentally different mechanism by which LOC is induced in SEVNI, namely rapid cortical failure secondary to catastrophic irreversible and fatal cerebral hypoperfusion (28, 30, 44). In SEVNI, brainstem injury and death occur at a delayed interval after LOC because brainstem tissue exhibits substantially greater resilience to anoxic conditions than the cerebral cortex (10, 11). Importantly, interpretation of reflex persistence in cattle must also account for interspecies neuroanatomical differences. In primates, voluntary motor control is predominantly pyramidal, such that cortical lesions produce profound contralateral motor deficits. In domestic species, however, the pyramidal system is comparatively less developed, and motor control relies more heavily on extrapyramidal pathways and brainstem nuclei, including the substantia nigra and red nucleus (4, 6). As a result, cortical dysfunction in cattle does not necessarily abolish motor output or brainstem-mediated reflex activity. Following SEVNI, upper motor neuron disinhibition may therefore preserve or even accentuate certain reflexive responses despite established LOC. These species-specific differences further caution against equating persistence of brainstem reflexes with ongoing consciousness.

The origin of a substantial portion of current international guidelines for the assessment of LOC following slaughter without stunning appears to be influenced by an experimental study by Verhoeven et al. (45). The influence of this study on international guidelines is understandable, given the relative scarcity of EEG-based investigations of slaughter without stunning in cattle to validate the use of specific indicators. The Verhoeven study examined whether the loss of menace response, corneal and eyelid reflexes correlated with EEG patterns interpreted as indicative of LOC in calves following slaughter without stunning. Importantly, the findings themselves confirm that cortical LOC occurs before the loss of palpebral and corneal reflexes (brainstem indicators), as both reflexes persisted for prolonged intervals after EEG-defined LOC. These observations are fully consistent with the neurobiological framework outlined above.

The emphasis on brainstem indicators for slaughter without stunning may also reflect a broader and longstanding problem in the literature on religious slaughter, namely the frequent conflation of LOC with brain death (44). Several studies have employed isoelectric EEG patterns (46–49), which signify brain death, or indicators of severe brainstem dysfunction such as pupillary dilation (50), absence of corneal and palpebral reflexes (51), and loss of rhythmic breathing (52), as proxies for LOC when estimating time to LOC following slaughter without stunning. Such approaches fail to recognize that LOC occurs as soon as cerebral blood flow and pressure is substantially compromised, well before brainstem failure, and is mediated primarily by rapid cortical dysfunction rather than brainstem anoxia. This conceptual error has been explicitly highlighted in a recent systematic review examining time to LOC following slaughter without stunning (44), yet it appears to have been carried forward into international guidelines that predate this review.

The historical development of behavioral indicators is likely linked to stunning-based slaughter methods. The extent of neural disruption produced by stunning methods such as captive bolt, electrical current, or gas exposure cannot be directly observed and therefore necessarily relies on animal-based behavioral indicators to confirm that LOC has occurred. Behavioral indicators therefore emerged as proxies to assess stun efficacy, with their importance heightened by the documented occurrence of ineffective stuns and the need for reliable detection of stun failure (51, 53–60). Animals subjected to repeated stun attempts experience significant suffering, underscoring the importance of reliable indicators in these systems.

By contrast, when performed correctly, SEVNI involves a visible and anatomically precise transection of the major vessels supplying blood to the brain. In fact, the WOAH guidelines explicitly recommend severing both carotid arteries and ensuring “*continuous, rapid blood flow following incision”* (Section 7.5.20), implicitly emphasizing the importance of visual wound inspection to confirm effective vessel transection (35). Since the carotid arteries are the primary source of cerebral cortical perfusion in cattle, when continuous, high-volume blood flow is observed, irreversible collapse of cerebral perfusion is directly verifiable and rapid cortical failure and, therefore, LOC are highly likely to occur (28–30, 44, 61–65). Importantly, visual confirmation of continuous, high-volume blood flow from the transected carotid arteries is not a behavioral indicator; rather, it reflects the physiological conditions of rapid loss of cerebral blood flow and pressure, consistent with inevitable and irreversible LOC, thereby reducing reliance on behavioral proxies. It has been suggested that limited collateral flow from the vertebrobasilar system may transiently persist after SEVNI (66, 67). More recent studies indicate this to be negligible and certainly insufficient to maintain or restore cortical function once the carotid circulation is opened (28–30).

Because indicators of LOC are intended for real-time use in commercial slaughter environments, their scientific validity cannot be considered independently of their operational feasibility, their reliability under high-throughput conditions, and any associated occupational safety implications. Loss of the menace response and eye tracking merit conceptual consideration as indicators of LOC in SEVNI, as both depend on intact cortical processing and their absence therefore provides a direct indication of cortical dysfunction and LOC. However, despite their sound neuroanatomical basis, assessment of the menace response and voluntary eye movements requires a calm environment with an unstressed animal and substantial operator expertise to reliably distinguish cortically mediated responses from reflexive blinking or eye movements, a distinction that can be challenging under high-throughput slaughter conditions.

In both conventional slaughter and SEVNI, reliance on reflex testing to assess LOC requires extensive operator training, calibration, and auditing frameworks to ensure consistency and reliability, yet remains subject to significant inter-observer variability (68). Furthermore, unlike conventional slaughter where animals are typically rendered motionless for ~5–15 s after a successful stun, menace response and reflex testing immediately after SEVNI necessitate close physical approach to the animal during a period in which residual reflexive or spinal mediated motor activity, such as kicking or jerking, may still occur (51, 69) thereby introducing avoidable occupational safety risks (41, 70). Taken together, in the context of SEVNI, these practical and safety considerations highlight the value of non-behavior-based indicators that reflect physiological inevitability and can be confirmed rapidly and reliably from a safe distance, while allowing clear, unobstructed visualization of continuous, high-volume blood flow from the transected carotid arteries.

Importantly, while visual wound inspection confirms the conditions leading to LOC, it does not directly measure cortical function, and like behavioral indicators and EEG, serves as an indirect proxy of cortical function. These limitations reflect broader challenges in animal research for both conventional slaughter with stunning and religious slaughter, where direct measurement of consciousness is not feasible. This represents an area for future investigation, including the application of artificial intelligence to integrate multimodal data such as facial expression, slaughter technique, and confirmation of effective carotid transection, alongside modeling of cerebral perfusion and cardiovascular dynamics (28). Furthermore, establishing parameters surrounding the slaughter technique and expected anatomical outcomes, including visual depictions or digital modeling, would provide a foundation for standardized training and competency-based assessment with demonstrable interobserver reliability. In the interim, visual confirmation of continuous blood flow remains a physiological proxy and should be considered within the broader framework of LOC assessment.
